# Base excision repair of ionizing radiation-induced DNA damage in G1 and G2 cell cycle phases

**DOI:** 10.1186/1475-2867-7-15

**Published:** 2007-09-24

**Authors:** M Ahmad Chaudhry

**Affiliations:** 1 Department of Medical Laboratory and Radiation Sciences, University of Vermont, Burlington, VT 05405, USA

## Abstract

**Background:**

Major genomic surveillance mechanisms regulated in response to DNA damage exist at the G_1_/S and G_2_/M checkpoints. It is presumed that these delays provide time for the repair of damaged DNA. Cells have developed multiple DNA repair pathways to protect themselves from different types of DNA damage. Oxidative DNA damage is processed by the base excision repair (BER) pathway. Little is known about the BER of ionizing radiation-induced DNA damage and putative heterogeneity of BER in the cell cycle context. We measured the activities of three BER enzymes throughout the cell cycle to investigate the cell cycle-specific repair of ionizing radiation-induced DNA damage. We further examined BER activities in G2 arrested human cells after exposure to ionizing radiation.

**Results:**

Using an *in vitro *incision assay involving radiolabeled oligonucleotides with specific DNA lesions, we examined the activities of several BER enzymes in the whole cell extracts prepared from synchronized human HeLa cells irradiated in G1 and G2 phase of the cell cycle. The activities of human endonuclease III (hNTH1), a glycosylase/lyase that removes several damaged bases from DNA including dihydrouracil (DHU), 8-oxoguanine-DNA glycosylase (hOGG1) that recognizes 7,8-dihydro-8-oxo-2'-deoxyguanosine (8-oxoG) lesion and apurinic/apyrimidinic endonuclease (hAPE1) that acts on abasic sites including synthetic analog furan were examined.

**Conclusion:**

Overall the repair activities of hNTH1 and hAPE1 were higher in the G1 compared to G2 phase of the cell cycle. The percent cleavages of oligonucleotide substrate with furan were greater than substrate with DHU in both G1 and G2 phases. The irradiation of cells enhanced the cleavage of substrates with furan and DHU only in G1 phase. The activity of hOGG1 was much lower and did not vary within the cell cycle. These results demonstrate the cell cycle phase dependence on the BER of ionizing radiation-induced DNA damage. Interestingly no evidence of enhanced BER activities was found in irradiated cells arrested in G2 phase.

## Background

Cells have developed multiple DNA repair pathways to protect themselves from different types of DNA damage. Oxidative DNA damage is processed by the short-patch base excision repair (BER) pathway. Depending on the structure of broken DNA ends, oxidative damage may be repaired by long-patch BER pathway. The nucleotide excision repair (NER) pathway appears to play a secondary role in the repair of these lesions, whereas recombination and translesion synthesis occur as mechanisms of damage tolerance [1-3]. Hydroxyl radicals affect DNA either by oxidizing its bases or by generating single strand breaks. Oxidized bases are processed through the action of BER machinery of the cell. Human endonuclease III (hNTH1) acts on oxidative pyrimidine lesions and catalyzes the hydrolysis of the *N*-glycosidic bond (*N*-glycosylase activity) subsequently incising apurinic/apyrimidinic, AP site (AP lyase activity) via β-elimination [4]. The remaining phosphodeoxyribose residue at the 5'-side of the break is removed by an AP-endonuclease or another phosphodiesterase activity and the repair is completed by the sequential action of a DNA polymerase and a DNA ligase. The cleavage of the DNA backbone at the apurinic/apyrimidinic (AP), or abasic, site is mediated by AP endonuclease (hAPE) activity. Human hAPE1 hydrolyzes the phosphodiester backbone immediately 5'of an AP site, generating an abasic deoxyribose 5-phosphate that is released by a 5'-deoxyribophosphodiesterase (dRPase) or 5'-exonuclease [5,6]. The human 8-oxoguanine-DNA glycosylase (hOGG1) recognizes the oxidative DNA lesion 7,8-dihydro-8-oxo-2'-deoxyguanosine (8-oxo-G). The terminal ends are modified by a 3'-phosphatase or 3'-phosphodiesterase, a reaction which is performed by hAPE1, before DNA polymerase β can fill in the correct nucleotide [7].

Genome integrity is protected by surveillance mechanisms known as DNA checkpoints. When activated by DNA lesions or replication blocks, these checkpoint pathways initiate DNA repair and delay cell cycle progression in order to prevent replication and segregation of damaged DNA molecules [8]. DNA damage-induced cell cycle checkpoints are a crucial mechanism for genomic stability [9]. Checkpoints ensure the proper execution of sequential events of the cell cycle so that cells containing damaged DNA do not proceed into S phase or into mitosis [10]. Cells exposed to ionizing radiation characteristically activate cell cycle checkpoints, resulting in cell cycle arrest [11]. The delay in progression through the cell cycle occurs at several checkpoints [8]. Delays in G1, S, and G2 have been described after exposure of cells to ionizing radiation [12,13]. These delays presumably provide time for repair of DNA damage. When checkpoint arrest control is compromised, initiation of S phase or mitosis occurs despite cellular damage leading to genetic instability. Arrest in G1 is thought to prevent aberrant replication of damaged DNA and arrest in G2 allows cells to avoid segregation of defective chromosomes. Studies have addressed the variations of radiosensitivity and endogenous cellular factors during cell cycle progression [14]. After exposure to ionizing radiation, the most radiosensitive cell stages are mitosis and the G1/S interface. High-LET radiation fails to slow progression through S phase but causes a more profound block in G2 phase than that observed after low-LET radiation [15].

By several indications it appears that DNA repair may vary throughout the cell cycle. In earlier studies unscheduled DNA synthesis was measured at different phases of the cell cycle after UV irradiation and was found to be very low in mitosis and highest in G2, S, and M phase cells [16]. UV-induced DNA repair endonuclease activity was examined in human fibroblasts and also found to be very low in mitosis [17,18], whereas no major variation in the incision activity of this enzyme was detected in other cell cycle phases [17,18]. It is shown that there are differences in the induction or rejoining of radiation-induced DNA single-strand breaks (SSBs) as a function of cell-cycle [19]. There is very little information available regarding the BER of ionizing radiation-induced DNA damage in the cell cycle context. The putative heterogeneity of BER in specific cell cycle phases has not been well studied in human cells. The present study was undertaken to investigate the cell cycle-specific repair of ionizing radiation-induced DNA base lesions and to determine BER activities in radiation-induced G2 arrested cells.

## Results

### Processing of DNA lesions furan, DHU and 8-oxo-G in G1 and G2 phases of the cell cycle

**Figure 1:**
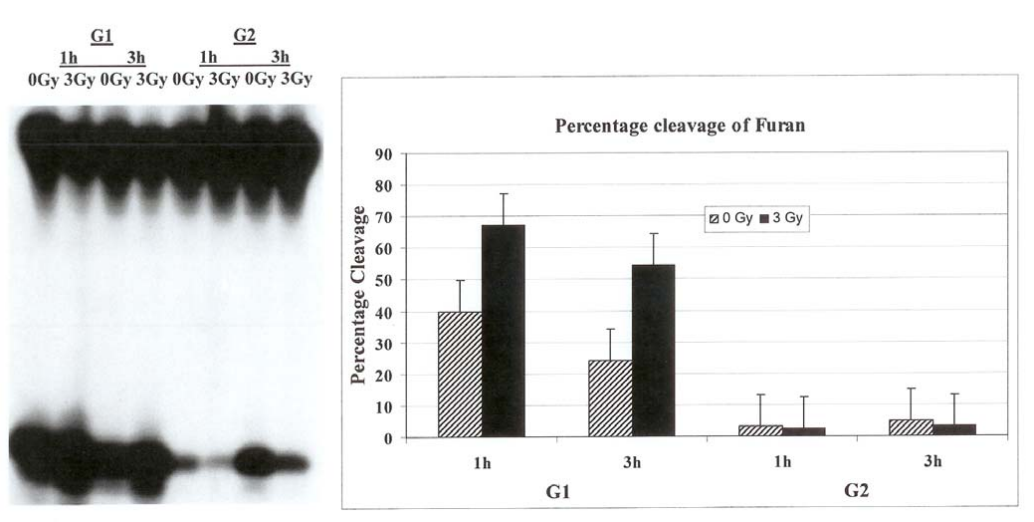
Incision of oligonucleotide containing furan lesion. HeLa cells were synchronized and Flow cytometer analysis confirmed their G1 or G2 status. The labeled oligonucleotide was incubated with cell extracts prepared from synchronized G1 or G2 HeLa cells either irradiated or not irradiated. The incision products on the gel were quantified with PhosphorImager to determine percentage cleavage.

HeLa cells were synchronized in G1 and G2 phases of the cell cycle as described in the materials and methods. The flow cytometry analysis confirmed that the cells were in G1 or G2 phase (data not shown). In the first set of experiments, synthetic oligonucleotide containing a single furan lesion was radiolabeled and incubated with cell extracts prepared from G1 or G2 synchronized HeLa cells either irradiated with 3 Gy or sham controls. The cells were incubated at 1 or 3 h at 37°C after irradiation. The cleavage at the lesion site was monitored after the separation of products on a polyacrylamide gel and autoradiography. There were marked differences in the cleavage patterns of furan lesion from the DNA. The activity of human apurinic/apyrimidinic endonuclease (hAPE1) that acts on abasic sites including synthetic analog furan was much higher in G1 compared to G2 as evidenced by an enhanced cleavage at the lesion site (Fig. 1). The percent cleavage of oligonucleotide substrate with furan lesion in G1 was as high as 40% while it was less than 5% in G2 phase. The investigation of the effect of irradiation on the enzymatic processing of furan lesion in the G1 and G2 synchronized cells revealed that the radiation treatment enhanced the activity of furan removal from the DNA in the G1 cell cycle phase (Fig. 1). Ionizing radiation did not seem to have a drastic effect on the incision activity of furan lesion in cell extracts prepared from G2 enriched cells (Fig. 1).

**Figure 2:**
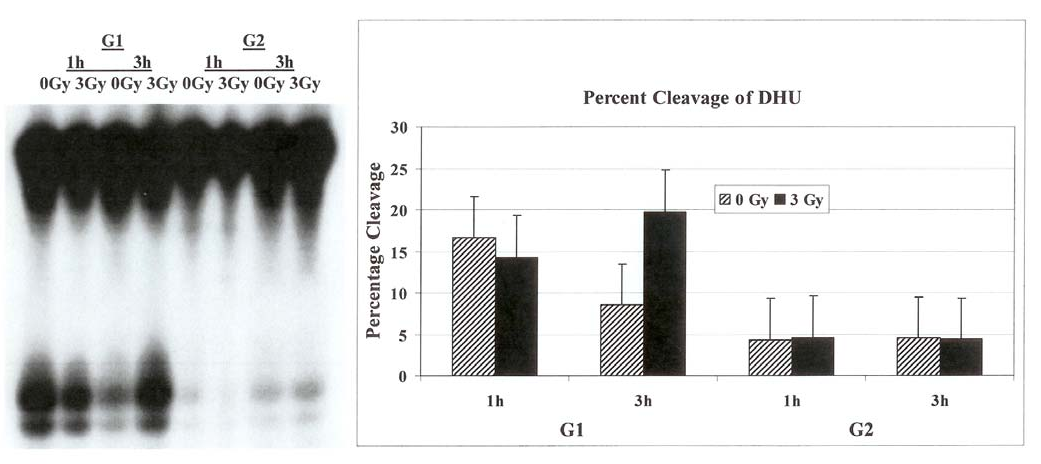
Cleavage of oligonucleotide containing DHU lesion. The labeled oligonucleotide was incubated with cell extracts prepared from synchronized G1 or G2 HeLa cells either not irradiated or irradiated with 3 Gy. The cleavage products on the gel were quantified with PhosphorImager.

The examination of cell extracts prepared from G1 or G2 phase synchronized HeLa cells either irradiated with 3 Gy or not irradiated to cleave radiolabeled synthetic oligonucleotides containing a single DHU lesion identified marked differences in the cleavage patterns of DHU lesion from the DNA between the two cell cycle phases. The activity of human endonuclease III (hNTH1), a glycosylase/lyase that removes several damaged bases from DNA including DHU, was higher in G1 as compared to G2 as evidenced by an enhanced cleavage at the lesion site (Fig. 2). The percent cleavage of furan substrate in G1 was as high as 15% while it was less than 5% in G2. We then investigated the effect of irradiation on the enzymatic processing of DHU lesion in the G1 and G2 synchronized cells. The radiation treatment resulted in a decrease in the activity of hNTH1 to remove DHU lesion from the DNA in the G1 cell cycle phase after one hour (Fig. 2) but this activity was enhanced after 3 hours (Fig. 2). No appreciable differences in the activity to remove DHU were noticed in G2 enriched cells after irradiation (Fig. 2).

**Figure 3:**
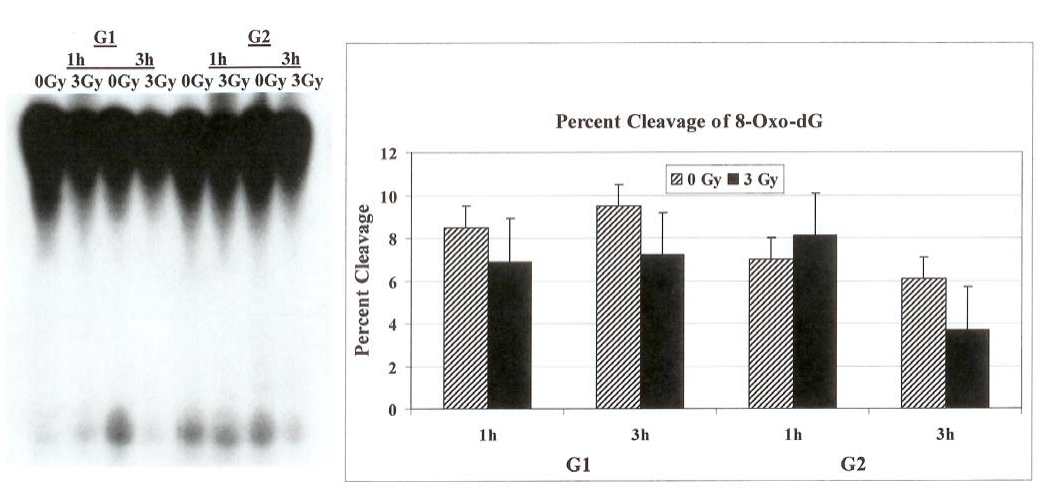
Incision of oligonucleotide with 8-Oxo-G DNA lesion. The labeled oligonucleotide was incubated with cell extracts prepared from synchronized G1 or G2 HeLa cells either irradiated or not irradiated. The cleavage products on the gel were quantified with PhosphorImager to determine percentage cleavage.

Radiolabeled oligonucleotide containing a single 8-oxo-G lesion was incubated with cell extracts prepared from synchronized, irradiated or controls HeLa cells. The overall activity of 8-oxoguanine-DNA glycosylase (hOGG1) that recognizes 7,8-dihydro-8-oxo-2'-deoxyguanosine (8-oxoG) lesion was lower in both G1 and G2 cell cycle phase as compared to furan or DHU removal. The cleavage patterns of 8-oxo-G lesion from the DNA did not differ between the two cell cycle phases (Fig. 3). The investigation of the effect of radiation on the enzymatic processing of 8-oxo-G lesion in the G1 and G2 synchronized cells did not identify any alterations in the activity of 8-oxo-G removal from the DNA in both phases of the cell cycle either after one or three hours (Fig. 3).

**Figure 4:**
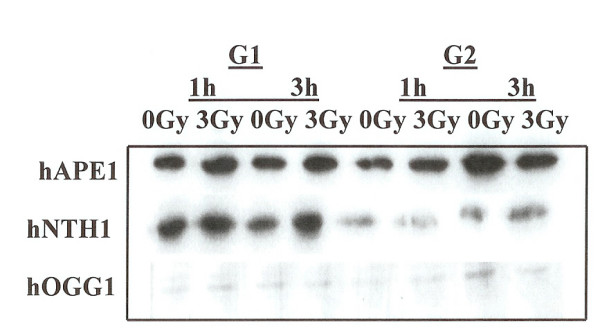
Western blot analysis of the cells synchronized in G1 or G2. The expression of hAPE1, hNTH1 and hOGG1 was examined in G1 and G2 phase cells. Cells were irradiated with 3 Gy and the expression of these proteins was monitored at 1 hour and at 3 hours post irradiation.

In order to assess if altered protein levels of hAPE1, hNTH1 and hOGG1 in G1 and G2 phases of the cell cycle were responsible for alterations in the repair activities of these enzymes, we monitored the cellular levels of these proteins in G1 and G2 enriched cells. The cells synchronized in G1 and G2 were collected to prepare protein extracts. After electrophoresis and blotting, these were probed with antibodies against hAPE1, hNTH1 and hOGG1. Fig. 4 shows that hNTH1 levels were lower in G2 phase as compared to G1 phase. The levels of hAPE1 and hOGG1 remained unchanged in the two phases. The overall expression level of hOGG1 was lower as compared to hAPE1 and hNTH1 (Fig. 4). We next asked the question if irradiation of G1 or G2 enriched cells lead to alteration in the protein levels of hAPE1, hNTH1 and hOGG1. The synchronized cells in G1 and G2 were irradiated with 3 Gy and after 1 hour and 3 hours the levels of hAPE1, hNTH1 and hOGG1 were monitored (Fig. 4). The radiation treatment did not cause any appreciable changes in the expression levels of these proteins in both cell cycle phases.

### BER activities in radiation-induced G2 phase arrested cells

**Figure 5:**
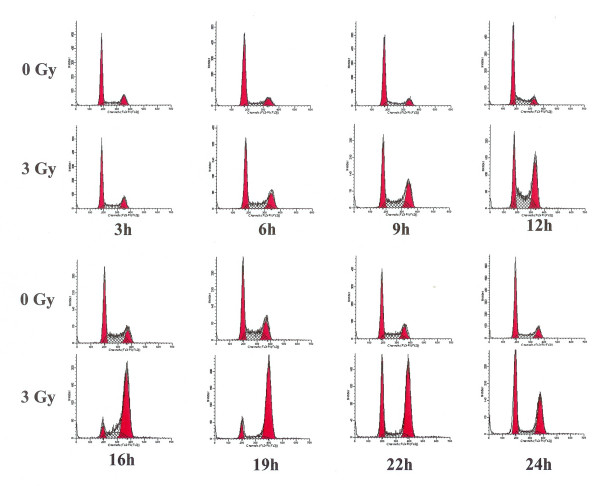
Cell cycle distribution of HeLa Cells after radiation exposure. Cells were irradiated with 3 Gy and samples were taken after 3 h, 6 h, 9 h, 12 h, 16 h, 22 h, and 24 h for flow cytometry analysis. The cell cycle distribution of irradiated cells was compared to unirradiated cells collected at the same time points. The left peek in each case represents cells in G1 and the right peek represents cells in G2 phase.

**Figure 6:**
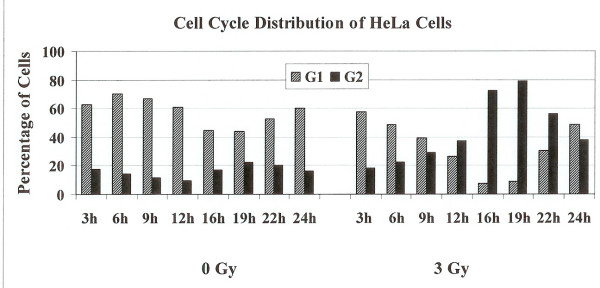
Percentage of cells in G1 or G2 phases in unirradiated or 3 Gy irradiated cells collected at various time points.

**Figure 7:**
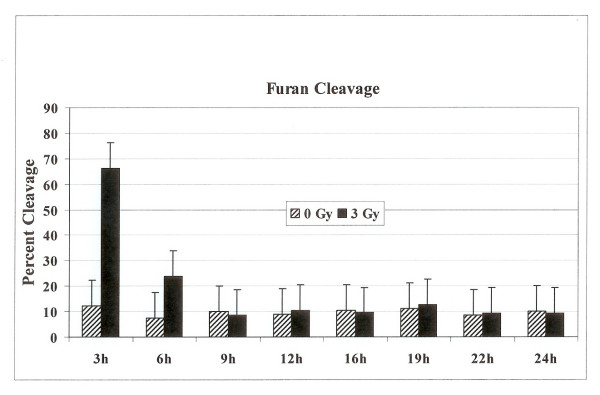
The percentage cleavage of oligonucleotide substrate containing furan lesion. The radiolabeled oligonucleotide substrate was incubated with cell extracts prepared from either unirradiated or 3 Gy irradiated cells collected at various time points. The reaction products were separated on polyacrylamide gel and the incised fragments were quantified to calculate the percentage cleavage at the furan site.

**Figure 8:**
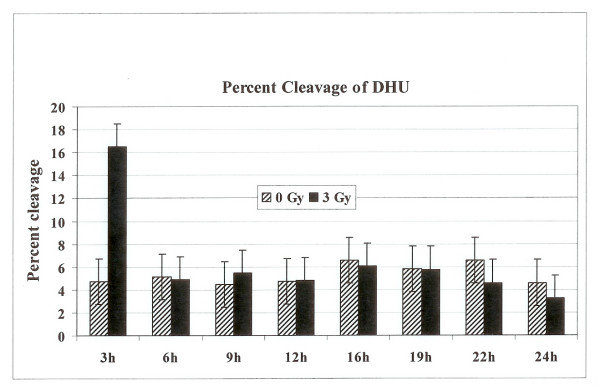
The percentage cleavage of oligonucleotide substrate containing DHU lesion. The radiolabeled oligonucleotide substrate was incubated with cell extracts prepared from either unirradiated or 3 Gy irradiated cells collected at various time points. The reaction products were separated on polyacrylamide gel and the incised fragments were quantified to calculate the percentage cleavage at the DHU site.

**Figure 9:**
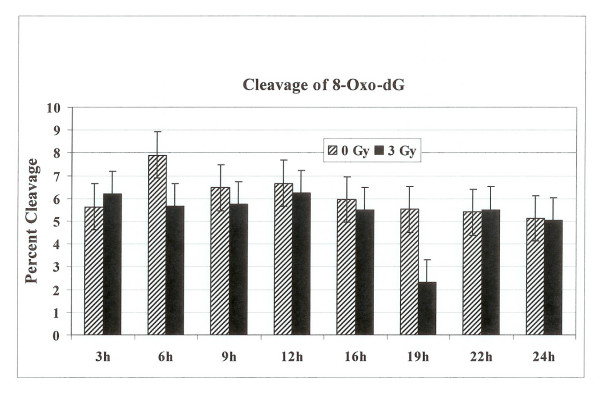
The percentage cleavage of oligonucleotide substrate containing 8-Oxo-dG lesion. The radiolabeled oligonucleotide substrate was incubated with cell extracts prepared from either unirradiated or 3 Gy irradiated cells collected at various time points. The reaction products were separated on polyacrylamide gel and the incised fragments were quantified to calculate the percentage cleavage at the 8-Oxo-dG site.

We next examined the BER activities in irradiated cells arrested in the G2 phase of the cell cycle. Exponentially growing HeLa cells were either mock irradiated or irradiated with 3 Gy of γ-radiation. Cells were harvested at 3 h, 6 h, 9 h, 12 h, 16 h, 22 h and 24 h post irradiation and subjected to flow cytometery analysis to assess their distribution in the cell cycle. The irradiated cells started to accumulate in the G2 phase after 12 hours of treatment and continued to accumulate in G2 phase until 19 hours post irradiation time (Fig. 5), where greater than 80% of the cells were in G2 (Fig. 6). After that time cells started to come back to G1 phase. No such accumulation of unirradiated control cells in the G2 phase was observed (Fig. 5). In order to examine the BER activities in the G2 arrested cells we investigated the capabilities of cell extracts prepared from irradiated cells at 3 h, 6 h, 9 h, 12 h, 16 h, 22 h and 24 h post irradiation to cleave oligonucleotide substrate containing a furan lesion, a substrate for hAPE1 activity. About 70% of the oligonucleotide substrate was cleaved at the furan site from cell extracts prepared at 3 h after irradiation (Fig. 7) suggesting that hAPE1 was activated during that time. This repair activity was declined by 6 h post irradiation (Fig. 7). No differences in hAPE1 activity were seen in irradiated cells at later time points, including the time when cells were arrested in G2 phase (12–19 h), as compared to controls. A very similar pattern for the repair of DHU lesion in the irradiated cells was observed (Fig. 8). The highest-level cleavage of DHU containing substrate happened at the 3 h time point in cell extracts from radiation treated cells. There was no difference in the DHU cleavage in irradiated versus unirradiated cells at all the later time points examined. Once again there was no enhanced DHU cleavage at times when irradiated cells were arrested in G2 phase. Finally we examined the capacity of cell extracts from irradiated cells to incise oligonucleotide containing 8-Oxo-G lesion (Fig. 9). No enhancement in the cleavage pattern of 8-Oxo-G substrate was observed in irradiated cells at all the time points investigated as compared to the unirradiated cells.

## Discussion

### Cell cycle specific expression of BER activities

We examined the activities of human endonuclease III (hNTH1), 8-oxoguanine-DNA glycosylase (hOGG1) and AP endonuclease (hAPE1) in G1 and G2 synchronized human HeLa cells using an oligonucleotide incision assay. Overall the enzymatic activities of hAPE1 and hNTH1 were higher in the G1 compared to the G2 phase of the cell cycle (Figs. 1 and 2). The activity of hOGG1 was much lower and did not vary within the cell cycle (Fig. 3). The low cleavage frequency of substrates containing 8-oxoG was seen even after optimizing experimental conditions. This could be due to low OGG1 expression in these cells. The Western blot analysis of the three repair proteins showed that hNTH1 was expressed at higher levels in G1 phase as compared to G2 phase (Fig. 4). There was no difference in the expression of hAPE1 and OGG1 in G1 and G2 phases. The OGG1 was expressed at very low levels. These results demonstrate the cell cycle phase dependence on the base excision repair of DNA damage. Several studies have analyzed the expression of DNA repair genes throughout the cell cycle. Investigation of the relationship between the cell cycle and *APE1 *expression in murine fibroblasts showed that transcription peaks in early S phase [20]. The expression of hAPE1 is also cell cycle dependent and increases during the G1 phase [21]. Similar studies with synchronized cells showed that the expression of hNTH1 is regulated during the cell cycle with increased transcription during early and mid S-phase [22]. The upregulation of other BER proteins in coordination with the S phase has been reported. The expression of *N*-alkyl-purine-DNA glycosylase (*MPG*) and uracil-DNA glycosylase increases during the G1 phase of the cell cycle, and decreases after mitosis [21]. Other studies showed that human uracil-DNA glycosylase (*UNG*) was increased in late G1/early S phase [23]. Cell cycle-dependent expression of the human MutY homolog, hMYH, an adenine-specific DNA glycosylase has also been reported [24]. The levels of hMYH increased during progression of the cell cycle and reached maximum levels in S phase compared to early G1. Similar results were obtained for PCNA [24].

In contrast, expression levels of hOGG1, TDG DNA glycosylases, O^*6*^-methylguanine-DNA methyltransferase (MGMT) gene, and the RPA4 genes do not vary with cell cycle [21]. The OGG1 gene expression and its enzymatic activity in cultured fibroblast cell lines did not vary during the cell cycle [25]. There were no notable changes in expression of hOGG1 or the human MutT homolog, MTH1, throughout the cell cycle [24]. It has been shown that OGG1 is a microtubule-associated protein facilitating the movement and redistribution of cytoplasmic OGG1 pools during interphase and mitosis and in response to oxidative DNA damage [26]. The observed cell cycle dependent differences might reflect distinct roles of individual BER proteins in mutation avoidance. Our data is in agreement with the published literature. Contrary to other reports we did not see alterations in the expression of hAPE1 and such affect could be cell type specific.

Coordinated expression of key DNA repair genes with the cell cycle appears to be a general phenomenon that applies to a number of genes, but it is not universal. The examination of nucleotide excision repair activity (including its coupling to transcription) was found to be similar throughout the cell cycle [27,28]. The apparent logic of cell cycle-regulated DNA repair expression (removal of mutagenic and replication-blocking damage ahead of replication forks) requires further exploration. Cell cycle-linked changes in the expression levels of DNA repair genes is one method that cells could use to reduce repair at normal bases and thereby preventing mutations [29,30].

### BER after irradiation in G1 and G2 cell cycle phases

The capacity to repair DNA lesions could be different in the different phases of the cell cycle. We investigated the effect of irradiation on the activities of hAPE1, hNTH1 and hOGG1 repair proteins in G1 and G2 phase synchronized cells. The radiation treatment in G1 phase enhanced the activity of hAPE1 but no such effect was observed in G2 phase (Fig. 1). This could be due to low enzymatic activity seen in G2, although the hAPE1 protein levels did not alter in two cell cycle phases (Fig. 4). The examination of hNTH1 in cell extracts prepared from G1 enriched control and irradiated cells to cleave oligonucleotide substrate with DHU lesion showed that hNTH1 activity in unirradiated cells was slightly declined at 3 hours time point (Fig. 2). The unirradiated cells were probably progressed in the cell cycle accounting for these differences. During extended incubation times hNTH1 activity was enhanced after irradiation in G1 cell cycle phase (Fig. 2). The induction of hNTH1 activity after IR exposure could also be due to IR transiently preventing cell cycle progression. There was no difference in the activity of hNTH1 in irradiated vs. unirradiated G2 phase cells. This could be due to reduced expression of hNTH1 in G2 phase (Fig. 4). The percent cleavage of furan was greater than DHU in the G1 and G2 phases (Figs. 1 and 2). The irradiation of cells enhanced the cleavage of furan and DHU only in G1 phase. The activity of hOGG1 was much lower and did not vary within the cell cycle after exposure to ionizing radiation. These results demonstrate the cell cycle phase dependence on the base excision repair of ionizing radiation-induced DNA damage.

Previous studies have shown that there was little variation in endonuclease activity during G1, S or G2 cell-cycle phases of UV irradiated cells [18]. In the synchronized cells, little variation in repair activity was discerned as cells transited from early G1 through late G1 and early S [18]. Other studies reported that there was an increase in the number of cells in G1 phase after UV exposure and it was correlated with repair during that time [31]. The examination of nucleotide excision repair (NER) and transcription coupled repair in synchronized and UV irradiated Chinese hamster ovary (CHO) cells in G1 and G2 phases identified no major differences [32]. Recent studies on synchronized Hela cells in G1 and S phases found that the S phase cells repaired DNA interstrand crosslinks more efficiently than G1-phase cells [33]. Our data has shown that there is a marked difference in BER activities in irradiated G1 and G2 enriched cells.

### BER in radiation-induced G2 arrested cells

Cell cycle delays after exposure to DNA damaging agents are well known. Such checkpoints would prevent cell division and subsequent transmission of damaged DNA to daughter cells. In response to DNA damage, cell survival can be enhanced by activation of DNA repair mechanisms and of checkpoints that delay cell cycle progression. It has been suggested that check points in G1 and in G2 allows time for DNA repair [34]. Based on the current literature it is not possible to prove the authenticity of this claim. Circumstantial evidence has suggested that there may be a relationship between the G2 delay and the DNA damage repair [35].

BER pathway deals with the ionizing radiation-induced DNA damage. The measurement of BER activities during the radiation-induced G2 delay has not been reported. We examined the distribution of cells in each phase of cell cycle, 3 to 24 h time period after γ-irradiation. The radiation treatment arrested cells in G2 phase of the cell cycle (Figs 5 and 6). The examination of hAPE1 (Fig. 7), hNTH1 (Fig. 8) and OGG1 (Fig. 9) did not identify any evidence of enhanced repair activities when cells were delayed in G2 phase. Previous studies have indicated that cells arrested in G2 phase exhibited higher repair endonuclease activity to incise 8-oxo-G than unirradiated cells [36]. It was suggested that the radiation-induced enhanced repair activity might be a feature of radiation-induced G2 arrest. We did not observe any differences in the incision of 8-oxo-G lesion in G1 or G2 irradiated cells nor we saw such enhanced repair in G2 arrested cells. Our data indicates that OGG1 is expressed at very low levels in HeLa cells and there is no difference in its expression levels in G1 or G2.

Kao *et al *attempted to demonstrate DNA repair during the G2 delay. Using an assay based on bromodeoxyuridine (BrdU) incorporation at discrete foci in the nucleus, these authors suggested that DNA repair activity occurs during the radiation-induced G2 delay [37]. Recently it was shown that DNA damage checkpoint proteins RPA, Rad9, and ATR were recruited to base damage induced by UV and acetoxyacetylaminofluorene in G1 and S phases of the cell cycle [38]. We expected that the delayed G2 phase of irradiated HeLa cells might be associated with increased levels of repair endonuclease activity directed against synthetic oligonucleotides where one strand of the duplex DNA contained furan, DHU or 8-oxo-G, major radiation induced products. Our data has clearly indicated that there is no enhancement of BER activities of at least hAPE1, hNTH1 and OGG1 enzymes in radiation treated G2 arrested HeLa cells.

## Conclusion

The results reported here demonstrate the cell cycle specific expression of BER activities. The enzymatic activities of hAPE1 and hNTH1 were higher in the G1 compared to the G2 phase of the cell cycle. The activity of hOGG1 was much lower and did not vary within the cell cycle. The hNTH1 enzyme was expressed at higher levels in G1 phase as compared to G2 phase. There was no difference in the expression of hAPE1 and OGG1 in G1 and G2 phases. The OGG1 was expressed at very low levels in both cell cycle phases.

The capacity to repair DNA lesions could be different in the different phases of the cell cycle. The investigation of the effect of irradiation on the activities of hAPE1, hNTH1 and hOGG1 repair proteins in G1 and G2 phases identified that the radiation treatment in G1 phase enhanced the activity of hAPE1 but no such effect was observed in G2 phase. These results demonstrate the cell cycle phase dependence on the base excision repair of ionizing radiation-induced DNA damage.

Cells exposed to ionizing radiation characteristically activate cell cycle checkpoints, resulting in cell cycle arrest. It is presumed that these delays provide time for the repair of damaged DNA. However, the measurement of BER activities during the radiation-induced G2 delay has not been reported before. The examination of hAPE1, hNTH1 and OGG1 in the present study did not identify any evidence of enhanced repair activities when cells were delayed in G2 phase.

## Methods

### Cell culture, irradiation and flow cytometry

HeLa cells were cultured at 37°C in DMEM medium (Invitrogen, Inc.) with 5% fetal calf serum, 1% penicillin and streptomycin. Exponentially growing cells were irradiated with 3 Gy of γ-radiation using a ^137^Cs irradiator (Nordion International ISO 1000, Model B) at dose rate of 0.12 Gy/sec. at the Red Cross facility (Burlington, Vermont). The control cells did not receive any radiation and were mock irradiated. After irradiation the cells were incubated at 37°C for various periods of times before collecting cells for flow cytometry analysis, preparing cell extracts or isolating proteins for western analysis. To collect a population in G2, the cells were synchronized using a thymidine-aphidicolin double block [39,40] and as previously described [41]. Ten hours after release from aphidicolin block when the majority of the cells were in G2, cells were treated with 3 Gy of γ-radiation. After one hour or three, the cells were harvested for flow cytometry and for whole cell extracts preparation. Control cells were treated identically except that the radiation treatment was omitted. Nocodazole blocking was used to synchronize cells in the G1 phase as described previously [41]. Samples were taken for flow cytometric analysis to confirm synchrony. G1 phase cells were collected and irradiated with 3 Gy. After further incubation for 1 and 3 hours, samples were taken from control and irradiated cells for flow cytometric analysis and for whole cell extract preparation. Flow cytometric analysis was performed to measure the DNA content and cell cycle distribution in the cells as described by Vindelov and Christensen [42]. Cell cycle analysis was performed within 2 h of staining on a Becton Dickinson FACScan flow cytometer. Ten thousand events were collected for each sample and the data was analyzed by using the MODFIT™ version 2.0 cell cycle analysis software (Becton Dickinson).

### Preparation of cell extracts

Approximately 10^7 ^cells were lysed in 400 μl of buffer containing 0.07 M HEPES pH 7.6, 0.4 M NaCl, 0.001 M EDTA and 10% glycerol and cell lysates were immediately frozen in liquid nitrogen. Upon thawing, triton X-100 was added to a final concentration of 0.2%. The Bradford assay was used to determine the protein concentration.

### Incision assay

The sequences of all the oligonucleotides used in this study are shown in Table 1. [see additional file 1] The amount of cell extracts and the time required to obtain maximal cleavage for each substrate was determined in a series of experiments (data not shown). ^32^P (γATP) labeled oligonucleotide containing a single furan was annealed with complementary strand (Table 1, A and D) and incubated at 1 nM with 0.015 μg of whole HeLa cell extracts in 50 μl reaction mixture containing 45 mM HEPES (pH 7.8), 70 mM KCl, and 1 mM DTT at 37°C for 10 minutes. Sample, 10 μl were removed from reaction mixture for cleavage analysis. Oligonucleotide containing single dihydrouracil (DHU) or 7,8-dihydro-8-oxo-deoxyguanine (8-oxo-G) was annealed with complementary strand (Table 1: B, C and D) and incubated at 1 nM with 0.85 μg (for DHU) or 2 μg (for 8-oxo-G) of whole HeLa cell extracts. The reactions were carried out in 50 μl buffer containing 25 mM HEPES (pH 7.9), 50 mM KCl, 2 mM DTT, and 2.5 mM MgCl_2_, and 10 nM or 25 nM competitor oligonucleotide for DHU or 8-oxoG respectively, at 37°C for 20 minutes. Samples, 10 μl were removed for cleavage analysis. In all the incision assays the labeled oligonucleotides were incubated in buffer only without the addition of cell extracts and no background cleavage was observed. All the experiments were independently repeated three times and standard error were included in the figures.

### Analysis of cleavage products by gel electrophoresis

Reactions were terminated on ice by adding an equal volume of the loading buffer (98% of formamide, 0.1% of bromophenol blue, 0.1% of xylene cyanole FF and 10 mM EDTA). The samples were subjected to electrophoresis in a 12% denaturing polyacrylamide gel with 7 M urea and TBE buffer (SequeGel Sequencing System Kit, National Diagnostics, Atlanta, GA). After electrophoresis, the gel was dried and analyzed by autoradiography or PhosphaImage Quantity One 4.21 Personal Molecular Imager FX (Bio-Rad Laboratories, Hercules, CA) using Quantity One^® ^software (Bio-Rad Laboratories, Hercules, CA). A double band after oligonucleotide cleavage was seen representing hydrolysis and β-elimination catalysis products.

### Western analysis

The whole cell lysates were boiled to denature the proteins. The proteins were separated on a 12% SDS-polyacrylamide gel and transferred to polyvinylidene difluoride membrane (Millipore) using a semi-dry blotter (W.E.P. Company, Inc.). After blocking overnight with 5% nonfat dry milk solution in PBS-T (PBS with 0.25% Tween 20) for hAPE1 and 10% nonfat dry milk in PBS for NTH1 and OGG1, the membranes were incubated overnight with anti-human NTH1 or OGG1 antibodies (recombinant Fab generously provided by Dr. Susan Wallace), or with anti-human hAPE1 (1:1000 dilution, Novus Biologicals) at 4°C. After washing (3 × 30 min., 5% or 10% nonfat dry milk in PBS-T), the membranes were incubated for 2 h with peroxidase-conjugated anti-mouse IgG peroxidase conjugate (1:10,000 dilution, Sigma). The protein antibody complexes were visualized by ECL plus (GE) according to the manufacturer's protocol. Chemiluminescence signals were captured directly by Fluor-S MAX2 MultiImager (Bio-Rad) or by autoradiography.

## Competing interests

The author(s) declare that they have no competing interests.

## Authors' contributions

MAC searched the literature, designed the analysis, drafted and prepared the manuscript.

## Supplementary Material

Additional file 1 Sequence of oligonucleotides. DNA sequence of the oligonucleotides. *F*, *8 *and *D *represent the lesions of Furan, 8-OxoG and DHU respectively. Sequence D serves as a complimentary sequence to A, B and C. Sequence E is the non-lesion sequence. Click here for file
