# Supplementary figures and images for: Base excision repair of ionizing radiation-induced DNA damage in G1 and G2 cell cycle phases
Source: Cancer Cell Int. 2007 Sep 24;7:15. doi: 10.1186/1475-2867-7-15 (PMC2063494; doi:10.1186/1475-2867-7-15)

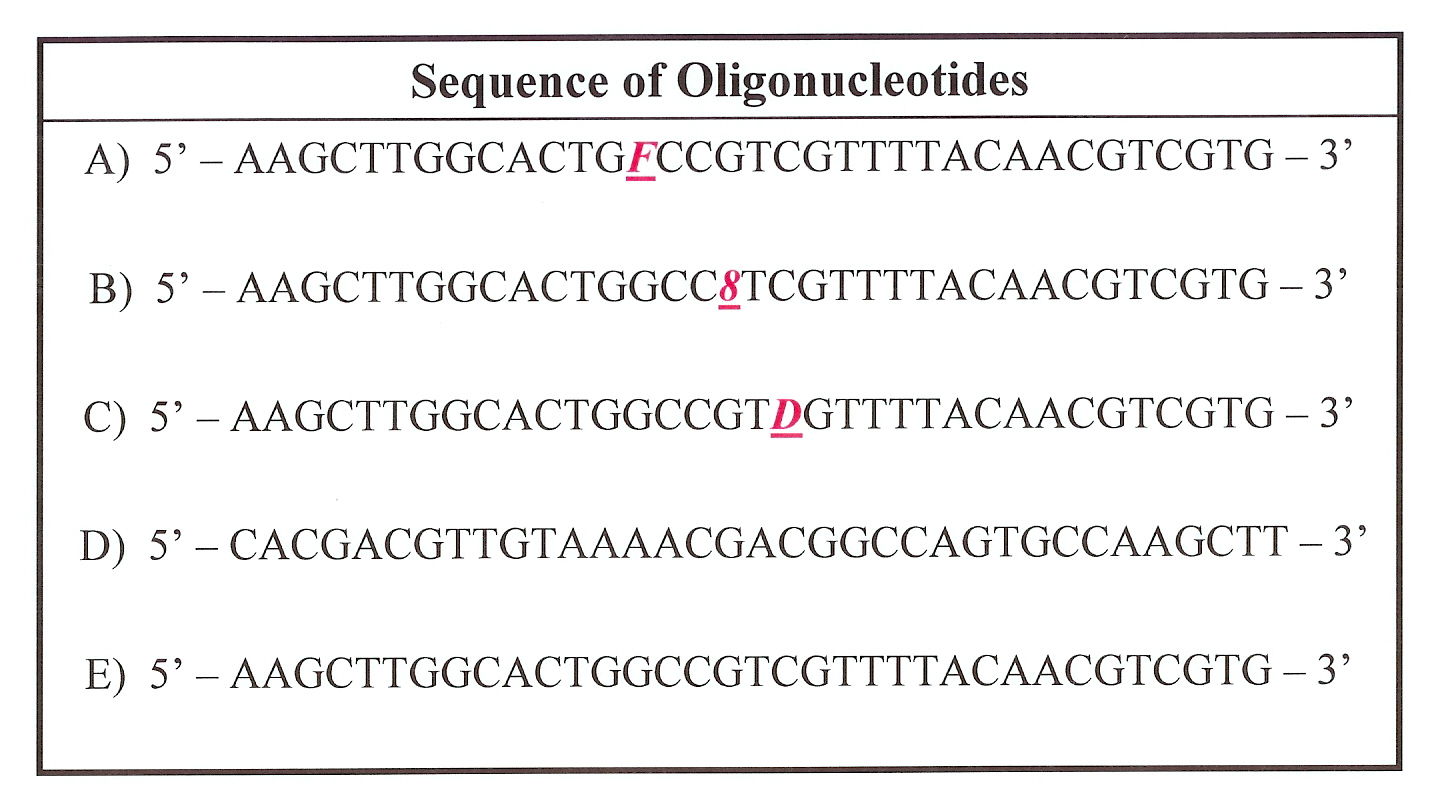

Supplement: Additional file 1 — Sequence of oligonucleotides. DNA sequence of the oligonucleotides. F, 8 and D represent the lesions of Furan, 8-OxoG and DHU respectively. Sequence D serves as a complimentary sequence to A, B and C. Sequence E is the non-lesion sequence. [file 1475-2867-7-15-S1.jpeg]
